# Supplementary material for: European System for Cardiac Operative Risk Evaluation II and Liver Dysfunction
Source: Biomedicines. 2025 Jan 10;13(1):154. doi: 10.3390/biomedicines13010154 (PMC11762396; doi:10.3390/biomedicines13010154)
Supplement: Supplementary file 1 [file biomedicines-13-00154-s001.zip › biomedicines-3387768-supplementary.pdf]

In Table S1 it can be seen that the bootstrapping and cross-validation of the initial models maintains the initial discrimination parameters in the bootstrap model and cross-validation models, having almost the same values in the bootstrap model, while in the cross-validation models, the Nagelkerke R2 index, Slope, discrimination (D) and unreliability (U) indexes are slighty increased, along with an increase of the Overall Quality (Q) and g-index. The Somers's D (Dxy) index is variable across all methods, although showing the same approximate value (approximately 0.6).

Table S1. Validation of Cox-proportional hazard multivariable model which used MELD and EUROSCORE predictive variables.

**Parameter/Method**	**Original Model**	**Bootstrap (*n* = 1000)**	**10-fold CV**	**Repeated 10-fold CV (N=100)**
Dxy	0.6060	0.5994	0.6182	0.6066
R2	0.2164	0.1822	0.3463	0.3217
Slope	1.0000	0.8893	1.9431	1.9530
D	0.0564	0.0446	0.1631	0.1548
U	-0.0026	0.0135	0.1452	0.0948
Q	0.0590	0.0311	0.0179	0.0599
g	0.6162	0.5112	0.9827	1.0801

CV = Cross-validation, *n* = Number of repetitions
